# Case report of Ganser syndrome in a 14-year-old girl: another face of depressive disorder?

**DOI:** 10.1186/1753-2000-6-6

**Published:** 2012-02-01

**Authors:** Michel Spodenkiewicz, Olivier Taïeb, Mario Speranza, Marie-Rose Moro, Anne Révah-Levy

**Affiliations:** 1 INSERM U669, Paris-Sud Innovation Group In Adolescent Mental health, 97, boulevard du Port Royal, 75014 Paris, France; 2 Department of Child and Adolescent Psychiatry, Université Paris 13, Centre Hospitalier Avicenne, 125, rue de Stalingrad, 93009, Bobigny, France; 3 EA 4047 UVSQ, Centre Hospitalier de Versailles, Service de Pédopsychiatrie, Le Chesnay, France; 4 Department of Adolescent Psychiatry, Université Paris Descartes, Centre Hospitalier Cochin, Maison des adolescents, AP-HP, 97, boulevard du Port Royal, 75014 Paris, France; 5 CSPTA, Centre Hospitalier Victor Dupouy, 69, rue du Lieutenant-colonel Prud'hon, 95107, Argenteuil, France

**Keywords:** Ganser syndrome, Adolescence, Depressive disorder

## Abstract

The Ganser syndrome is rare in children and in adolescents. A case of the Ganser syndrome in a 14-year-old girl, with three of the four essential features, is presented. After rapid resolution in two weeks, Ganser symptoms reappear seven months later accompanied, this second time, by previous depressive symptoms. This report raises the possibility of the Ganser syndrome as a manifestation of a depressive disorder in adolescence.

## Background

In 1898, Ganser [1] delivered a paper entitled "Concerning an unusual hysterical confusional state" in which he described prisoners who developed transitory symptoms of mental illness, characterized by disturbed consciousness, hallucinations, sensory changes of a hysterical kind, and characteristic answers to questions. Ganser termed these answers to questions *vorbeigehen*, i. e. passing by or beside the point, or giving approximate answers to questions within the patient's range of knowledge. Enoch and Trethowan [2] state that the four essential clinical features of the Ganser syndrome are: approximate answers, somatic conversion symptoms, clouding of consciousness, and visual or auditory pseudohallucinations.

Controversy has always surrounded the precise nosological status of the Ganser syndrome. Several authors such as Cocores et al. [3] have insisted on the relationship with malingering disorders, schizophreniform disorders, a hysterical dissociative state, or finally organic or toxic confusional states. Modern classifications subsume this syndrome under the general heading of unspecified dissociative disorders as in DSM-IV (300.15), and it is generally accepted in the literature that the Ganser syndrome is a dissociative disorder. However Sigal et al state [4] that the underlying mechanisms are still under discussion, and the organic and/or psychotic nature of this entity is still controversial. More recently, Fink and Taylor suggested that the Ganser syndrome could be one of the forms of catatonia [5].

In children and adolescents, the Ganser syndrome appears to be especially rare. Nine cases have been reported by Whitlock [6] in 1967, Nardi and Di Scipio [7] in 1977, Adler [8] in 1981, Burd and Kerbeshian [9] in 1985; Dabholkar [10] in 1987, Adler and Touyz [11] in 1989, Apter et al. [12] in 1993, Miller et al. [13] in 1997 and the last by Refaat et al. [14] in 2002). These cases met at least one of Ganser's original symptoms, the only feature being the approximate answers. Comorbid context was frequent: schizophrenia (Whitlock [6]; Nardi and Di Scipio [7]), major or minor head injury (Adler [8]; Adler and Touyz [9]; Miller et al. [13]), bipolar disorder (Apter et al. [12]), behavioral disorder (Apter et al. [12]), Gilles de la Tourette syndrome (Burd and Kerbeshian [9]; Refaat et al. [14]), and atypical pervasive developmental disorder (Burd and Kerbeshian [14]). The course of these cases also varied from rapid remission, which is the most frequent outcome, to chronic course with symptoms still present after many years follow-up (Adler and Touyz [11]; Refaat et al. [14]).

The lack of reports and information about its course made it worthwhile reporting the case of a 14-year-old girl who presented a Ganser syndrome, resolved in two weeks and followed seven months later by a relapse of the same syndrome, preceded by a major depressive episode. The possibility of the Ganser syndrome as a co-morbid condition or an expression of depressive disorder in adolescence will be discussed.

## Case presentation

C is a 14-year-old girl who came to the emergency room in a bizarre state, with altered consciousness. It was just after spring break. Her parents came with her.

### Background information

C was living with her parents and her 16-year-old sister. C comes from an upper middle class Catholic family. Both parents are teachers in an elementary school. The only psychiatric background in the family concerned one aunt (one of her mother's sisters) who had been undergoing treatment for depression for two years with a complete remission. C was a premature child (33 weeks) at birth but her psychomotor development was normal. No psychological or medical problems have been reported in the early childhood. C had her menarche when she was 13 years old. Neither C nor her sister had medical or psychiatric previous record. No evidence of recent traumatic events have been found in the family. Her learning skills have always been average. She was described by her parents as too serious and highly concerned by her school results while not experiencing any academic difficulties. She was also described as being shy and dependent.

### Recent context

Before Easter vacation, one of C's aunts (the wife of one of her father's brothers) died of lung cancer three weeks after she was diagnosed but the parents didn't inform C right away because she was taking her school exams. At the burial, C was very sad but there were no pathological manifestation. Her behavior and reactions were considered normal.

The Easter vacation started a few days later. The first week of vacation C went to Prague with her school. Every pupil stayed in a Czech host family. No problem occurred during this week. Soon after she came back, her parents discovered that she lied to them. They had asked her to bring back crystal glasses for which they had given her some money. C came back with the glasses, but she did not tell her parents they had been a present from the family she visited, and she used the money for other purposes. C was very upset when her parents discovered the truth, even if they didn't consider this event relevant.

The day after a discussion concerning her untruthfulness, C suddenly presented the first symptoms, in the form of insomnia, logorrhea, anxiety with incoherent discourse and amnesia of recent events, such as her aunt's death and the recent holiday in Prague. She said that she was her dead aunt, she wondered about her origins: "I am the daughter of my aunt", she said to her father: "You are not my father". While watching a film on television, she said she recognized places where she spent her childhood. Her parents took turns to stay with her night and day. She would sleep only four hours a night. There was neither agitation nor aggressiveness but several fluctuations a day from mutism to logorrhea with incoherent ideas about her origins.

The following days, no change occurred but her parents still hoped she would recover spontaneously. She was unable to return to school. Then the parents referred her to a physician who sent her to our emergency room.

### Initial medical and psychiatric assessment

In the emergency room, C was quiet. She only complained about sleep disturbance. She also answered questions with approximate answers. When asked who she was, she said: "Anne". When asked where she was, she replied: "At my place". When asked who were the people who came with her, she replied: "My uncle and my aunt". She also gave wrong answers to questions involving simple arithmetic, for example "three plus three equals seven". C wondered about her filiation: "What if my father was not my father? I may be Jacques Chirac's (*former president of France*) daughter or your daughter." There was no other delusion. Her discourse was diffluent, going from questioning her origins to her aunt's death and finally to the holiday in Prague. She also presented visual hallucinations. She was scared, pointing to snakes on the walls of the emergency room. Her behavior varied from one interviewer to another: with one, she could be confused giving approximate answers, with another, she could be mute and opposing. The clinician reported no other signs of disintegration of thought processes and of emotional responsiveness.

In view of the atypical presentation, an organic etiology was looked for. Physical and neurological examination results were normal. A computed tomographic (CT) head scan and a EEG showed no abnormalities. Blood test results were also normal and the drug screen was negative. There was no history of head injury or trauma, or other illness.

### The first hospitalization

C was therefore admitted to our child and adolescent psychiatric unit. The two first days of the hospitalization, C received 25 mg a day of cyamepromazine. She started sleeping normally again within 48 hours. She did not seem to suffer from hospital life. No behavioral disorder was reported. The first week, we observed fluctuations in the symptoms during the day, alternating phases of coherence with a good adaptation and phases of confusion with approximate answers. By the second week of hospitalization, stable recovery was complete. Delusions and hallucinations didn't last; she presented amnesia of the episode.

The psychological tests produced results within the normal range.

After staying 15 days in hospital, she came back home without treatment and was able to return to school.

### Follow-up

Until the end of the summer vacation C did not present any disturbances. In September, C went back to school for the beginning of the school year without any trouble. But in the beginning of November, after a school holiday, she started to cry a lot, with irritability, decreased concentration, loss of appetite, negative self perceptions, suicidal ideation, telling her parents she wanted to die, and never go back to school. She refused to consult her psychiatrist during this period and the general practitionner of the family advised to wait.

At the beginning of December, she presented insomnia, expressed intensive negative self-perception, refused to go to school and isolated herself at home. Her parents reported that at the end of December for a few days she seemed to be "in a numbed state, talking nonsense". On New Year's Eve, she suddenly started dancing on her own in the middle of the family meal, talking about another dancer dressed in black. Her parents, alarmed by this behavior and by the visual hallucinations, decided to bring her back to our emergency room.

### The second hospitalization

At the emergency room she arrived with a very sad look on her face, she only spoke when called upon to do so. She presented a delirious discourse on her filiations, and approximate answers; there were moments of disturbed consciousness and visual hallucinations. She though she was in "something like a cemetery". She cried a lot, and between periods of confusion she said she wanted to die. No other symptom of a psychotic disorder has been reported. No stress, drug consumption or any other precipitating factor have been found linked with this episode.

The next day, on the ward, the psychiatrist made the decision to treat her with fluoxetine at 20 mg/day, on account of the chronology that had been noted: appearance of a depressive state, in November, which was not treated, and deterioration of the depressive state, followed by appearance of Ganser symptoms (clouding of consciousness, approximate answers, and visual hallucinations). She also received 25 mg/day cyamepromazine at bedtime for the first three days. The Ganser symptoms improved from the start of the third week of fluoxetine administration; there was no further clouding of consciousness, no visual hallucinations, and depressive symptoms (insomnia, irritability, suicidal ideation and depressed mood) disappeared at the same time. She was hospitalized one month total and then returned to school.

### Outcome

Fluoxetine treatment was prescribed at 20 mg/day, but after two months C started to refuse to take any medication and stopped taking it. The school year ended uneventfully; there were no further disturbance. C continued with a combination of psychiatric follow-up and interpersonal psychotherapy for another year. No further episode occurred. Then C decided to end up to the follow-up.

## Conclusions

To our knowledge, this is the first case reported of recurrent Ganser syndrome in an adolescent. It is also the first report of a Ganser syndrome preceded by major depression symptoms in adolescence. In fact this case showed three of the four major features of the Ganser syndrome, i. e., the approximate answers, the clouding of consciousness and hallucinations. Following the rapid resolution of Ganser syndrome in two weeks, it reappeared seven months later, preceded by depressive symptoms, including depressed mood, decreased concentration, feelings of decreased self-worth, suicidal ideation, loss of appetite, and insomnia.

Some authors have reported affective disorders in the follow-up of patients with Ganser syndrome, but no one has presented recurrent Ganser symptoms and, in all instances, depressive or manic symptoms occurred after the resolution of the Ganser syndrome, except for one case described by Grieger and Clayton [15]. In adolescents, Apter et al [12] have described two brothers (15 and 16) with conduct disorder who developed the Ganser syndrome while in jail and awaiting trial; both subsequently developed symptoms of bipolar disorder. The elder brother presented psychotic depression six months later, followed after several weeks by hypomania. The younger brother also presented a period of depressive symptoms followed by a manic-like psychosis. In adults, Grieger and Clayton [15] described the case of a 25-year-old woman had been reported with a history of recurrent depression who presented major depression a few weeks after resolution of a Ganser syndrome. Haddad [16] reported a similar case of a 31-year-old man with a mild mental handicap who presented a Ganser syndrome resolved after one week but immediately followed by major depression. There are different psychodynamic hypothesis to explain the association between affective disorders and the Ganser syndrome. Apter et al. [12] have postulated that for a large number of patients, the development of severe regression and dissociation of the Ganser syndrome is facilitated by the presence of serious comorbidity, such as affective disorders. Haddad [16] states that the Ganser syndrome and depression are also considered as separate manifestations of a common underlying conflict. When it is no longer possible to avoid the unpleasant situation and the burden of responsibility by the dissociative defense, guilt and symptoms of depression appear.

In the present case, our first interpretation was the appearance of the Ganser syndrome enabled our patient to escape from the guilt about lying to her parents, via a dissociative response and fast resolution of symptoms, as in the classic descriptions of the Ganser syndrome. The recent death of the aunt should be emphasized, as it probably rendered the patient more vulnerable. But the way the case evolved alters this first interpretation, with a second phase where depressive symptoms appear over a few weeks before the reappearance of the Ganser symptoms. On account of this chronology of disturbances, the following question appears relevant: wasn't the first episode a co-morbidity or also the manifestation of a depressive disorder?

In a recent article, Pietsch and colleagues emphasized how diagnosing a depressive episode in adolescence is complicated [17]. A few other authors such as Moro [18] or Kirmayer and Groleau [19] state nosography has hard time in apprehending the depressive experience, and its multiple and varied expressions. Revah-Levy and colleagues have shown how the diagnostical instruments nowadays used to measure depression are not specific to adolescents, lack construct validity, and have limited or unknown reliability and validity in this age group [20]. Kessler [21] states there is controversy around the designation and identification of the polymorphic symptoms that might be significant in adolescent depression. McClellan and Werry [22] state that even the efficiency of antidepressants leads us to reconsider the syndromic differences between depression in young people and that classically diagnosed in adults. The difficulties encountered in catering for depression in adolescents point to the need to consider, as Brooks and Kutcher [23] or McClellan and Werry [22] state, that the depressive experience in adolescence as being outside categorical approach.

It is by examining the way the depressive experience was expressed by our patient that it was possible *a posteriori *to consider the Ganser symptoms as probable signs of a depressive experience that was not recognized at the time of the first hospitalization. It was treated, although at a late stage, at the time of the second hospitalization. The evolution with complete and definitive recovery of dissociative symptoms suggests that the first episode wasn't schizophrenia or an other delusional disorder. The idea of comorbidity in a patient without any previous antecedents is more unlikely due to the closeness of the two psychiatric events. However we cannot exclude this possibility.

With respect to clinical implications, it should be emphasized that, even though the Ganser syndrome is uncommon in children and adolescents, its recognition is necessary to distinguish this disorder from psychotic disorders, especially in adolescence. There is also always a need for physical investigations to exclude any possible organic factors and to identify the underlying disorder. In our case, the use of an antidepressant was effective in improving the mood and it is possible that by treating the depression it also reduced Ganser symptoms, which can be seen as manifestation of depression. Our case reinforces the view that children and adolescents with Ganser syndrome should be admitted for assessment and suggests the need for follow-up after apparent recovery. The risk of recurrence of Ganser syndrome should also be better known. We believe our case helps to clarify the outcome and prognosis of Ganser syndrome in adolescence by helping clinicians to become aware of the fact that the Ganser syndrome might be present in addition to a depressive disorder as a co-morbid condition or as another form of manifestation of depressive experience in adolescence.

## Competing interests

The authors declare that they have no competing interests.

## Authors' contributions

MS, OT, MAS and OT drafted the manuscript. ARL participated in collecting and discussing clinical data. MRM carried out clinical assessment and discussion. All authors read and approved the final manuscript

## Consent statement

Written informed consent was obtained from the patient for publication of this case report and accompanying images. A copy of the written consent is available for review by the Editor-in-Chief of this journal.
